# Echocardiographic predictors of exercise capacity and mortality in chronic obstructive pulmonary disease

**DOI:** 10.1186/1471-2261-13-84

**Published:** 2013-10-12

**Authors:** Mikkel Malby Schoos, Morten Dalsgaard, Jesper Kjærgaard, Dorte Moesby, Sidse Graff Jensen, Ida Steffensen, Kasper Karmark Iversen

**Affiliations:** 1 Department of Cardiology, Rigshospitalet, University Hospital Copenhagen, Blegdamsvej 9, 2100 Copenhagen, Denmark; 2 Department of Cardiology and Endocrinology, Hillerød Hospital, Hillerød, Denmark; 3 Department of Pulmonary and Infectious Diseases, Hillerød Hospital, Hillerød, Denmark

**Keywords:** COPD, 6MWD, Exercise capacity, Pulmonary artery pressure, Systolic dysfunction, Diastolic dysfunction, Tissue doppler, Global longitudinal strain

## Abstract

**Background:**

Chronic obstructive pulmonary disease (COPD) reduces exercise capacity, but lung function parameters do not fully explain functional class and lung-heart interaction could be the explanation. We evaluated echocardiographic predictors of mortality and six minutes walking distance (6MWD), a marker for quality of life and mortality in COPD.

**Methods:**

Ninety COPD patients (GOLD criteria) were evaluated by body plethysmography, 6MWD and advanced echocardiography parameters (pulsed wave tissue Doppler and speckle tracking).

**Results:**

Mean 6MWD was 403 (± 113) meters. All 90 subjects had preserved left ventricular (LV) ejection fraction 64.3% ± 8.6%. Stroke volume decreased while heart rate increased with COPD severity and hyperinflation. In 66% of patients, some degree of diastolic dysfunction was present. Mitral tissue Doppler data in COPD could be interpreted as a sign of low LV preload and not necessarily an intrinsic impairment in LV relaxation/compliance. Tricuspid regurgitation (TR) increased with COPD severity and hyperinflation. Age (p < 0.001), BMI (p < 0.001), DLCO SB (p < 0.001) and TR (p 0.005) were independent predictors of 6MWD and a multivariable model incorporating heart function parameters (adjusted r^2^ = .511) compared well to a model with lung function parameters alone (adjusted r^2^ = .475). LV global longitudinal strain (p = 0.034) was the only independent predictor of mortality among all baseline, body plethysmographic and echocardiographic variables.

**Conclusions:**

Among subjects with moderate to severe COPD and normal LVEF, GLS independently predicted all-cause mortality. Exercise tolerance correlated with standard lung function parameters only in univariate models; in subsequent models including echocardiographic parameters, longer 6MWD correlated independently with milder TR, better DLCO SB, younger age and lower BMI. We extended the evidence on COPD affecting cardiac chamber volumes, LV preload, heart rate, as well as systolic and diastolic function. Our results highlight lung-heart interaction and the necessity of cardiac evaluation in COPD.

## Background

Chronic obstructive pulmonary disease (COPD) is associated with increased pulmonary vascular resistance by hypoxic pulmonary arterial vasoconstriction, and by mechanical compression of the pulmonary vasculary bed due to air-way obstruction and air trapping [1-4]. Emphysema is associated with pulmonary alveolar and vascular destruction which leads to increased ventilation-perfusion mismatching with reduced diffusing capacity and to loss of pulmonary elastic recoil and parenchymal support for collapsible airways. This results in hyperinflation with elevated intrathoracic pressure, which is associated with reduced left ventricular end-diastolic volume, stroke volume and cardiac output, estimated by magnetic resonance [5]. Recently, Barr et al. showed that the extent of computer tomography measured emphysema and airflow obstruction are linearly related to impaired left ventricular filling, reduced stroke volume, and lower cardiac output without changes in left ventricular ejection fraction (LVEF) already in mild to moderate COPD, [6] and Watz et al. found a clear association between hyperinflation (inspiratory capacity to total lung capacity ratio - IC/TLC) and decreasing dimensions of all four cardiac chambers estimated by echocardiography for the entire spectrum of COPD severity [7]. Another readily available echocardiography estimate is peak triscupidal regurgitation, a proxy for pulmonary artery pressure (PAP). In COPD, hyperinflation, wedge, right atrial, and invasively measured PAP increase parallel during tachypnea and exercise [8]. PAP is independently associated with reduced exercise function in patients with severe COPD, who were evaluated for lung transplantation [9].

The aim of the present study was to determine if 1) the simple echocardiographic estimates of peak tricuspidal regurgitation and measures of impaired ventricular filling predict six minutes walking distance (6MWD) in patients with moderate COPD and 2) how more advanced echocardiographic pulsed wave tissue Doppler and speckle tracking variables relate to body plethysmography, 6MWD prediction and mortality in COPD.

## Methods

We included 101 COPD patients without history of cardiovascular disease and normal LVEF (>50%) from the outpatient clinic of the department of pulmonary diseases, Hillerød Hospital, Denmark. COPD diagnosis was according to GOLD diagnostic criteria [10] and a post-bronchodilator spirometry with FEV_1_/FVC < 0.70. GOLD class I (mild COPD) was defined by a forced expiratory volume during one second (FEV_1_) ≥80% of normal, GOLD class II (moderate COPD) by FEV_1_ 50–79% of normal, GOLD class III (severe COPD) by FEV_1_ of 30–49% of normal and GOLD class IV (very severe COPD) by FEV_1_ <30% of normal. Severity of airflow limitation was based on post-bronchodilator FEV_1_. We hypothesized that abnormalities in echocardiographic variables would be most overt in the most pulmonary ill patients, why the primary inclusion goal was to recruit participants in GOLD class II - IV. Patients were approached during outpatient clinic consultation, based on their most recent spirometry result. These values tend to fluctuate and especially borderline cases can re-categorize between measurements, why also a few GOLD class I patients were included. Patients in GOLD class IV were relatively more reluctant to participation or too ill with domestic oxygen therapy. After written consent, patients were tested on the same day by body plethysmography, transthoracic echocardiography and 6 MWD, in this order. Echocardiographic and pulmonary function measures were made at rest. One patient was excluded because of previous pulmonary lobectomy, 3 patients because of either missing body plethysmography or transthoracic echocardiography, 5 patients due to previous myocardial infarction and 2 patients due to congestive heart failure. This study was approved by the local ethics committee (H-A-2008-138).

### Transthoracic echocardiography

Transthoracic echocardiography was performed blinded to all other data, using a Vivid 7 ultrasound system (GE Healthcare). Images were obtained from the parasternal views (long axis and short axis), the apical four-chamber view and the subcostal view. All analyses were performed using dedicated software (EchoPAC, GE). Echocardiographic assessment was performed according to guidelines [11-13].

### Systolic function

LVEF was evaluated by Simpson’s biplane method, also yielding the body surface area (BSA) indexed LV volumes: stroke volume index (SVI) = left ventricular end-diastolic volume index (LVEDVI) – left ventricular end-systolic volume index (LVESVI). Tissue Doppler–derived mitral lateral annular systolic velocity (S’ LV) and longitudinal global strain (GLS LV) were evaluated using an automatic tracing algorithm with manual adjustment.

Two-dimensional speckle tracking was performed using a semiautomatic algorithm (Automated Function Imaging, GE Healthcare). Manual positioning of 3 points (2 annular and 1 apical) was performed in the 4-chamber apical projection, enabling the software to semi-automatically track the myocardium. The region of interest was adjusted to cover the thickness of the myocardium. Aortic valve closure was identified on continuous-wave Doppler recording through the aortic valve. Careful inspection of tracking and manual correction, if needed, was performed. The Automated Function Imaging algorithm allowed GLS to be calculated if at least 5 of 6 segments were sufficiently tracked. The algorithm then calculated overall GLS as the mean value [14]. Right ventricle (RV) systolic function was evaluated using tricuspid annular plane systolic excursion (TAPSE), tissue Doppler–derived tricuspid lateral annular systolic velocity (S’ RV), and longitudinal GLS RV tracing the RV free wall in the apical 4-chamber with manual adjustments. We also measured the peak systolic pressure gradient of the tricuspid regurgitation (TR) jet (mmHg).

### Diastolic function

Measurement of LV and RV diastolic function was assessed by pulsed Doppler of the mitral/ tricuspid inflow (early wave (E), atrial wave (A), E/A ratio, deceleration time (DT) and slope of the early transmitral/transtriscuspidal flow), tissue Doppler-derived diastolic early (e’) and late (a’) velocities of the lateral mitral/tricuspid annulus as well as tissue Doppler-derived isovolumetric contraction time (IVCT), isovolumetric relaxation time (IVRT), ejection time (ET) and the E/e’ ratio. Diastole is divided into relaxation, represented in part by IVRT, and diastolic filling, represented in part by deceleration time of the early transmitral/transtriscuspidal flow and ratio of the peak velocity of the early E-wave to atrial A-wave (E/A).

### Chamber volumes

Indexed left (LAVI) and right atrium (RAVI) volumes were assessed at the end of ventricular systole (largest volume) in the apical 4-chamber view. Indexed LV volumes (ml/m^2^) were determined during Simpson’s biplane method [13]. RV focused apical 4-chamber views (transducer adjusted to focus on the RV chamber, with the goal of maximizing chamber size) were used to obtain BSA indexed basal (RVD1I), mid cavity (RVD2I) and longitudinal dimension (RVD3I) at end-diastole.

### Lung function variables

Lung function variables were measured by spirometry and body plethysmography. Among the indexes used, diffusion capacity of carbon monoxide transferred from the alveoli to the capillary during a single breath (DLCO SB) was employed as a gas exchange functional index. Reduced DLCO SB is an index of loss of interalveolar septa as well as of restrictive pneumopathy. FEV_1_ was interpreted as a measure of respiratory capacity and classifies COPD severity. The ratio of FEV_1_ to the forced volume vital capacity (FEV_1_/FVC) is a measure of obstruction to exhalation. Total lung capacity (TLC) is the sum of functional residual capacity (FRC) and inspiratory capacity (IC). The ratio of IC/TLC and the determination of residual volume allows to judge upon the degree of lung hyperinflation. Values of RV <140% of predicted are indicative of mild, values between 140 and 170% of moderate, and values >170% of severe hyperinflation [15].

### Oxygen delivery index

The oxygen delivery index (DpO_2_) was calculated by the following equation, relying on pulse oximetry (SpO2): DpO2 = 1.34 × Hb conc. × Cardiac Index × (SpO2/100) ml/min/m2.

### Statistics

Continuous variables are presented as median and interquartile range (IQR) or mean and standard deviation (±SD). We used the Kruskal-Wallis test to explore significant differences in median values. Non parametric correlation was tested by Pearson correlation. Kaplan Meier curves and log rank test was used to estimate mortality differences according to GOLD class. All univariable predictors with *P* <0.1 were entered in multivariable linear and binary logistic regression analysis of exercise tolerance and all-cause mortality predictors. A P-value <0.05 was considered significant. Statistical testing was performed using the PASW Statistics software package, version 19 (SPSS, Inc., Chicago, IL, USA).

## Results

We included 90 COPD patients without previous ischemic heart disease, congestive heart failure, LVEF <50%, aortic stenosis or mitral insufficiency. The mean 6MWD was 403 ± 113 meters. Baseline characteristics in terms of age and gender were balanced and body plethysmography parameters were associated with GOLD classes as expected. Risk factors of underlying cardiac disease were unevenly dispersed. Frequencies of diabetes and dyslipidemia were very low, however 30–40% hypertensive patients were present in GOLD class II and III (Table 1).

**Table 1:** Baseline variables

	**GOLD class**				
	**I**	**II**	**III**	**IV**	***P***
N	5	35	41	9	
Age	73 (62–77)	70 (63–77)	66 (63–74)	67 (65–71)	0.740
Female (%)	3 (60.0)	22 (62.9)	27 (65.9)	2 (22.2)	0.205
Hypertension	1 (20)	13 (40.6)	15 (37.5)	1 (11.1)	0.309
Diabetes	1 (20)	3 (9.1)	1 (2.3)	0 (0)	0.038
Dyslipidemia	1 (20)	10 (30.3)	5 (12.5)	0 (0)	0.015
BMI	29 (23–29)	26 (23–30)	24 (21–26)	24 (17–29)	0.109
FEV1,% of predicted	84.8 (83–86.8)	57.6 (52.9–69.9)	40.1 (36.4–43.4)	25.8 (24.6–27.5)	<0.001
FVC,% of predicted	101 (92.5–109.6)	96.4 (82.8–107.8)	82.6 (71.1–89.1)	52.2 (49–63)	<0.001
FEV1/FVC,%	69 (65–74)	51 (44–60)	41 (35–45)	38 (35–41)	<0.001
IC,% of predicted	98.9 (95.8–121.8)	97.3 (87.7–109.8)	80.4 (65.3–96.4)	65.7 (56.1–67.2)	<0.001
TLC,% of predicted	105.9 (98.3–115.4))	122.7 (105.3–134.8)	126 (111.8–134.4)	128.6 (115.1–135))	0.127
Residual volume,% of predicted	102.9 (101.6–139.2)	155.5 (139.4–185.6)	190 (172–215.4)	241.8 (195.2–252.6)	<0.001
FRC_pleth_,% of predicted	106.7 (100.5–113.2)	146 (117–170.7)	167.2 (148–179.5)	185.9 (168.7–198.9)	<0.001
TLCO SB,% of predicted	75.1 (66.7–82.7)	52.6 (43.7–67.3)	40.7 (31–49.5)	30.2 (20.8–42.5)	<0.001
IC/TLC	0.40 (0.39–0.46)	0.34 (0.30–0.38)	0.27 (0.22–0.33)	0.22 (0.20–0.23)	<0.001
Hemoglobin g/L	130 (116–147)	137 (129–150)	140 (135–147)	142 (130–154)	0.641
Hemoglobin mmol/L	8.1 (7.2–9.1)	8.5 (8–9.3)	8.7 (8.4–9.1)	8.8 (8.1–9.5)	0.641
DpO_2_ ml/min/m^2^	281 (279–294)	326 (290–413)	295 (251–329)	366 (349–391)	0.007
SAT_rest_	95 (95–96)	96 (94–96)	95 (96–96)	93 (91–95)	0.048
SAT_min_	93 (89–95)	91 (87–93)	88 (86–91)	84 (80–85)	0.001
∆ SAT	2 (1–6)	4 (3–6)	7 (4–9)	9 (7–10)	0.003
6MWD	520 (450–575)	425 (355–510)	380 (310–450)	355 (325–375)	0.009
TR (mmHg)	27.8 (26.0–28.8)	29.1 (25.8–35.0)	33.2 (28.3–41.0)	40.5 (37.4–48.3)	0.001

*FVC* = forced volume vital capacity; *FEV*_*1*_ = forced expiratory volume in one second; *TLC* = total lung capacity; *FRC*_*pleth*_ = functional residual capacity; *IC* = inspiratory capacity; *DLCO SB* = Single breath diffusing capacity of the lung for carbon monoxide; *IC/TLC* = inspiratory capacity to total lung capacity ratio; *SAT*_*rest*_ = Saturation at rest; *SAT*_*min*_ = Minimal saturation during 6MWD; ∆ SAT = SAT_rest_ - SAT_min_; 6MWD = 6 minutes walking distance.

### Systolic function

All subjects had preserved LV ejection fraction 63.9% (± 8.8) and overall GLS was 18.4 (± 3.8). Stroke volume decreased and heart rate increased significantly with increasing COPD severity (Table 2). Right ventricular function in terms of TAPSE, S’RV and GLS RV were not associated with COPD severity. However, the basal right ventricular diameter tended to increase with COPD (Table 2). TR increased significantly with COPD severity in terms of FEV_1_ (r = -.416; P <0.001) and with hyperinflation and emphysema in terms of residual volume (r = .297; P = 0.004), function residual capacity (r = .322; P = 0.002) and IC/TLC (r = -.410; P <0.001)) and DLCO SB (r = -.367; P <0.001) as well as airway obstruction (FEV_1_/FVC) (r = -.373; P <0.001). Furthermore, we found a significant inverse relationship between GLS and DpO2 (r = -.322, P = 0.016).

**Table 2:** Systolic variables and ventricular volumes

	**GOLD class**				
	I	**II**	**III**	**IV**	***P***
Left systolic function					
LVEF	64 (59–67)	62 (58–68)	69 (65–76)	57 (57–57)	0.138
Heart rate	69 (65–76)	80 (74–85)	80 (75–86)	87 (85–88)	0.030
SVi (ml/m^2^)	22.0 (21.8–28.1)	22.9 (21.0–26.5)	19.4 (17.8–22.3)	20.7 (18.3–24.9)	0.001
CI (L/min^-1^*m^2^)	1.7 (1.6–1.8)	1.8 (1.6–2.0)	1.6 (1.3–1.8)	1.8 (1.7–2.1)	0.015
S’ (LV) (cm/s)	9 (7–9)	8 (7–9)	9 (7–11)	9 (7–10)	0.383
GLS (LV)	15.7 (15.3–17.5)	16.8 (14.8–20.2)	19.4 (16.4–22.4)	18.2 (14.4–19.2)	0.066
Right systolic function					
TAPSE	2.2 (2.2–2.3)	2.3 (2.1–2.5)	2.3 (2.1–2.6)	2.2 (2.1–2.4)	0.885
S’ (RV) (cm/s)	13 (10–14)	13 (12–14)	14 (13–15)	13 (12–14)	0.428
RVDi	18.3 (18.0–21.64)	20.0 (17.4–21.3)	20.4 (17.4–22.4)	22.3 (19.2–23.3)	0.164
GLS (RV)	26.2 (18.7–33.7)	23 (19.2–26.6)	23.8 (19.7–27)	22.8 (18–34.4)	0.968

*CI* = Cardiac index; *SVi* = stroke volume index; *TR* = Tricuspidal regurgitation; *GLS* = Global strain; *LVEF* = Left ventricular ejection fraction.

As hypertension was unevenly dispersed between GOLD class groups, we investigated the possible differences between patients with known hypertension and non-hypertensive patients in order to account for a possible afterload effect on ventricular function and volumes. We found no significant differences regarding GLS, LVEF by Simpson biplane as well as ventricular and atrial camber volumes.

### Diastolic function

Diastolic measures are shown by GOLD class (Table 3) and the comparison to reference values from guidelines publications is shown in Table 4. When applying the recommendations for evaluation of left ventricular diastolic function, assessed by e’, atrial volume, and mitral inflow patterns identified by E/A ratio and DT, [11] this COPD population with normal LVEF had a high incidence of diastolic dysfunction: 25 (34.2%) had normal function, 34 (40.8%) had diastolic dysfunction grade I (impaired relaxation pattern), 18 (20%) had diastolic dysfunction grade II (pseudonormalized flow), and 1 (1.3%) had diastolic dysfunction grade III (restrictive pattern).

**Table 3:** Diastolic variables

	**GOLD class**				
	**I**	**II**	**III**	**IV**	***P***
Left heart diastolic function					
LAi	21 (20–22)	18 (15–22)	18 (14–23)	14 (14–19)	0.402
Lateral Ee’ (LV)	9.42 (8.1–11.9)	8.66 (7.7–10)	8.92 (7.8–11)	6.9 (6.4–9.2)	0.451
Mitral E/A	0.91 (0.73–1.31)	0.80 (0.62–0.99)	0.85 (0.69–1.14)	0.85 (0.68–0.97)	0.360
DT (ms)	236 (215–292)	239 (205–254)	226 (173–250)	212 (176–246)	0.762
IVRT (ms)	90 (87–135)	87 (71–99)	79 (65–11)	77 (76–86)	0.891
Right heart diastolic function					
RAi	20 (14–21)	19 (15–22)	19 (15–27)	21 (19–24)	0.836
Lateral Ee’ (RV)	4 (3–4)	4 (4–5)	4 (3–5)	4 (4–4)	0.563
Tricuspidal E/A	1.2 (1.0–1.3)	1.1 (0.9–1.3)	1.1 (1.0–1.3)	1.2 (1.1–1.2)	0.813
DT (ms)	225 (205–232)	208 (171–243)	215 (169–272)	155 (153–229)	0.493
IVRT (ms)	76 (63–86)	86 (74–100)	92 (79–101)	93 (77–98)	0.497

**Table 4:** Diastolic variables in the overall population in comparison to reference values from normal populations from guidelines publications

	**Measured values**	**Reference values**
Left heart diastolic function		
E	80 (±18)	73 (± 19)[11]
A	90 (±23)	69 (± 17)[11]
E/A ratio	0.99 (± 0.55)	0.96 (± 0.18)[11]
Lateral e’ (cm/s)	9.2 (± 2.8)	12.9 (± 3.5)[11]
Lateral E/e’ ratio	9.1 (± 2.6)	6.7 (± 2.2)[11]
Lateral e’/a’ ratio	0.92 (± 0.42)	0.9 (± 0.4)[11]
IVRT (ms)	93 (± 23)	87 (± 7)[11]
DT (ms)	226 (± 50)	200 (±29)[11]
Right heart diastolic function		
E	55 (± 11)	54 (52–56)[12]
A	49 (± 11)	40 (38–41)[12]
E/A ratio	1.2 (± 0.25)	1.4 (1.4–1.5)[12]
Lateral e’ (cm/s)	11.9 (± 2.7)	14 (CI 13–14)[12]
Lateral E/e’ ratio	4.27 (± 2)	4 (CI 4–4)[12]
Lateral a’ (cm/s)	119.1(± 4.6)	13 (CI 12–14)[12]
Lateral e’/a’ ratio	0.66 (± 0.24)	1.2 (CI 1.1–1.3)[12]
IVRT (ms)	89 (± 19)	48 (CI 43–53)[12]
DT (ms)	210 (± 56)	174 (CI 163–186)[12]

### Exercise tolerance

Diastolic dysfunction was not significantly associated with exercise capacity; Normal: (median 425 IQR 355–480 m); grade I: (390 IQR 310–450 m); grade II: (420 IQR 290–550 m); grade III: (100 m) (P = 0.278)].

In multivariable linear regression analysis age, BMI, DLCO SB and TR were independent predictors of 6MWD distance. A multivariable model incorporating TR as the only independent heart function parameter predicting 6MWD increased the adjusted r^2^ value compared to a model with lung function parameters alone from 0.475 to 0.511 (Table 5).

**Table 5:** Univariable and multivariable predictors of distance (m) performance in 6 minutes walking test

	**Univariable predictors**		**Multivariable predictors**		**Multivariable predictors**	
			**Model 1**		**Model 2**	
	**β**	**P**	**β**	**P**	**β**	**P**
Age	-.305	0.004	-.406	<0.001	-.323	<0.001
Gender			.153	0.064		
BMI			-.649	<0.001	-.595	<0.001
FEV_1_	.305	0.004				
FEV_1_/FVC	.196	0.067				
IC	.272	0.010				
IC/TLC	.361	<0.001				
DLCO SB	.390	<0.001	.547	<0.001	.608	<0.001
SAT_rest_	.288	0.006				
TR	-.386	<0.001			-.239	0.005
E/e’	-.285	0.015				

BMI, gender, residual volume, functional residual capacity, total lung capacity and cardiac stroke volume index were also tested for univariable prediction but were insignificant.

Model 1 tests multivariable prediction of baseline variables (age, gender, BMI) and lung function variables only (FEV_1_, FEV_1_/FVC, IC, IC/TLC, TLC, RV, DLCO SB, SAT_rest_).

Model 2 tests echochardiographic univariable predictors of 6MWD (TR, CI, E/e’) added to model 1. Overall adjusted r^2^ values for model 1 and 2 are 0.475 and 0.511 respectively.

### All-cause mortality predictors

Median follow up time was 1387 (IQR 1122–1472) days. Increasing GOLD class was not consistently associated with increasing mortality: [GOLD class I: 1 (20%) event, GOLD class II: 11 events (32.4%), GOLD class III: 9 events (22%) and GOLD class IV: 4 events (50%), log-rank test *P* = 0.694]. GLS (LV) was the only independent predictor of all cause long term mortality among all baseline, body plethysmographic and echocardiographic variables (Table 6).

**Table 6:** All univariable predictors with P <0.1 are shown and were entered in a multivariable analysis of all-cause long term mortality

	**Univariable predictors**		**Multivariable predictors**	
	**HR (CI)**	***P***	**OR**	***P***
Male	2.90 (1.30–6.47)	0.009	2.94 (0.86–10.0)	0.085
FEV_1_/FVC	0.96 (0.93–0.99)	0.038		
DLCO SB	0.97 (0.94–0.99)	0.028		
GLS (LV)	0.78 (0.65–0.93)	0.006	0.82 (0.68–0.98)	0.034
6MWD	0.99 (0.99–1.00)	0.046		

All other variables in Table 1, 2, 3 were tested but non-significant.

## Discussion

The main results were the following; i) In patients with moderate to severe COPD and normal LVEF, GLS (LV) was the only independent predictor of all-cause long term mortality. ii) TR was an independent predictor of exercise tolerance in patients with moderate to severe COPD. iii) COPD severity is associated with significant changes in cardiac chamber volumes, heart rate and left ventricular filling properties.

### Mortality

All-cause mortality in GOLD class II & III was very similar, suggesting that the GOLD classification does not separate mortality risk properly. In univariable survival analysis, 6MWD, FEV1/FVC, DLCO SB and GLS (LV) correlated with mortality; in multivariable survival analyses however, mortality was predicted by GLS (LV) only and not by functional capacity, COPD severity or right ventricular structure and function (Table 6). We also found a paradoxical trend of higher GOLD class being associated with better LV systolic function as assessed by higher GLS (LV) values (Table 2). In a recent meta-analysis, normal ranges for GLS were established at a mean of 19.7 % (95% CI, 18.9 - 20.4 %) [16]. In comparison, values across all GOLD classes were lower in this population and in line with a recent study of sub-clinical LV dysfunction [17]. As GLS (LV) was inversely associated with mortality, this suggests that GLS (LV) may not mediate the association between COPD severity and mortality. If so, one may speculate that the data represent separate and independent pathophysiological contexts (left heart disease, functional capacity, lung dysfunction, mortality), meaning that the additive information by echocardiography is likely to be prognostic relevant information unrelated to COPD. This may also contribute to explain why right ventricular structure and functions were not significantly related to 6MWD and mortality. In contrast, the positive relationship of DLCO SB with better functional capacity and higher TR with shorter 6MWD confirm the association of COPD severity and functional capacity.

### Left heart systolic function

A recently emerged hyperinflation parameter is the inspiratory fraction (IC/TLC), which is associated with emphysema on high resolution CT [18] and independently predicts exercise capacity, [19] acute disease exacerbations, [20] and mortality in COPD patients [21]. We extended the evidence regarding the pattern of decreasing stroke volume and increasing heart rate with COPD severity and hyperinflation (Table 2 &7) – the 'shrinking heart’ in COPD. We also found the opposite relationship for heart rate and hyperinflation (Table 7). Previously, Barr et al. showed in a population-based cohort with mild to moderate COPD and without overt cardiovascular disease, that a greater extent of emphysema on CT scanning and more severe airflow obstruction (FEV_1_/FVC) were related to impaired left ventricular filling [6]. In a magnetic resonance study, patients with severe emphysema had approximately 40% reductions in SVI compared to control subjects [5]. Decreasing heart size is not only present in severe emphysema. Watz et al. showed a clear association between hyperinflation (IC/TLC) and decreasing volumes for all four cardiac chambers for the entire spectrum of COPD severity [7].

**Table 7:** Correlations of ventricular chamber volumes and measures of COPD severity and hyperinflation

		**HR**	**LVEDVI**	**SVI**	**CI**	**RVD1I**
FEV_1_ (%)	β	-.268	.265	.391	.202	-.218
	P	0.011	0.015	<0.001	0.065	0.061
IC/TLC	β	-.190	.339	.432	.339	-.335
	P	0.075	0.002	<0.001	0.002	0.003

There were no clear association of S’ or GLS (LV) with GOLD classes (Table 2). Unexpectedly, as cited above, we found a seemingly increasing dynamic heart with a tendency towards increasing S’ (LV) and GLS (LV) with increasing severity of COPD (Table 2). We see two possible explanations: Firstly, left ventricular filling pressure has an impact on left ventricular longitudinal systolic strain rate in preserved LVEF: lower filling pressures (usually considered normal filling pressures) are associated with higher peak systolic strain rate [22]. Secondly, these findings may also be explained by a hypoxia or baroreflex mediated increase in cardiac sympathetic nerve activity which will increase cardiac inotropy and chronotropy [23], which is supported by our findings of a significant. We found a significant inverse relationship between GLS and DpO2 (r = -.322, P = 0.016).

All of the above implies that increasing COPD severity and hyperinflation leads to reduced LV preload, entailing reduced stroke volume, but maintained cardiac output due to an increased heart rate. Alongside there are indices of a compensating increased longitudinal function with increasing COPD severity, possibly due to hypoxia induced and/or lower preload and filling pressures mediating increased ino- and chronotropy, in an otherwise longitudinal depressed left ventricle that predicts mortality.

### Right heart systolic function and pressure

Another finding of the present study was that greater peak TR independently predicted decreasing exercise function in moderate COPD, (Table 5) which is in line with previous reports, [9] although results are conflicting [24]. Recently, Boerritger et al. showed that only in COPD patients with severe pulmonary hypertension (invasively measured mPAP >40 mmHg found in rare 4% of patients), exercise capacity is limited because of an exhausted circulatory reserve [25]. This possibly indicates that TR in this population with moderate to severe COPD is a marker, rather than a cause of decreased exercise capacity. The feeble but consistent increase of TR with measures of COPD severity and hyperinflation did not result in depressed right long-axis ventricular function, as S’ RV and TAPSE did not differ from normal individuals [12] and TAPSE, S’RVand GLS RV were not associated with lung function parameters (Table 2). Yet, this was at the expense of a slightly dilated right ventricle associated with hyperinflation (Table 7), possibly due to increased right ventricular filling pressures, as mean IVRT RV was clearly increased compared to normal subjects (Table 4). Our finding of RV dilatation with a lowered LV size with COPD severity and hyperinflation fits in a mechanism of increased RV afterload by increased pulmonary artery pressure (and hence increased TR), leading to LV preload reductions. Several studies have shown right ventricular hypertrophy in COPD, [24,26,27] supporting the concept of increased RV afterload. Yet here, our results are opposed to Watz et al. who found a decreasing RV size with hyperinflation [7]. The presented values of SVI and CI (Table 2) are in comparison to recent studies rather low for moderate to severe COPD [9].

### Left heart diastolic function

Unlike previous reports, we did not find decreasing E/A and increasing deceleration time of E with increasing hyperinflation [7] or COPD severity, [28] nor did we find decreasing E/A with increasing SPAP [29]. In our overall population, numerically E/A and DT was very similar compared to normal individuals [11]. However, abnormal left ventricular diastolic filling patterns, assessed by e’, LAVI, and mitral inflow patterns (E/A and deceleration time of E), were present in 66% of patients, which compares to a recently found 54% prevalence by Cuttica et al. We also found lower E/e’ values to be univariably associated with impaired functional capacity (Table 5), confirming previous results by Cuttica et al. [24]. Decreasing E/e’ with increasing COPD severity indicates lower left ventricular filling pressures, [11] which lends support to the concept of reduced preload in COPD. We also confirmed previous findings that left IVRT LV was unaffected by hyperinflation [7], which might indicate that LV diastolic dysfunction in patients with COPD seems not to be related to left ventricular distensibility (ie. stiff myocardium or intrinsic impairment in LV relaxation/compliance) but rather to a reduced preload.It is important to acknowledge the problem that mitral Doppler and TD data, as well as IVRT are all load-dependent and heart rate-dependent and therefore should be interpreted with caution, particularly in COPD with lower preload levels.

### Right heart diastolic function

We found no significant associations of tricuspid Doppler inflow variables (E, A, E/A) with lung function parameters, but the overall population had compared to normal individuals reduced e’, clearly elevated a’ and reduced e’/a’ (Table 4). Reduced e’/a’ may indicate reduced preload to the right ventricle or/and increased right ventricular filling pressure, also indicated by clearly prolonged IVRT RV (Table 4), which reinforces the concept of increased RV afterload.

### Limitations

Our study had several limitations. The design was cross sectional, making firm conclusions about causality impossible. Also, we did not have a control group which necessitated the use of reference values from literature. This cohort mainly represents patients from GOLD class II and III and as a result, the findings of our study cannot necessarily be generalized. Finally, reliability of echocardiography measures of SPAP are disputed due to the difficulty in obtaining satisfactory echo images in a potential hyper inflated chest and rightward rotation of the heart, making visualization of the tricuspid valve and vena cava more difficult. In patients with severe emphysema, echocardiographic estimates of pulmonary artery pressures correlate very weakly with right heart catheterizations [30]. Also the low values of SVI/CI we found are possibly due to imaging difficulties.

## Conclusion

GLS predicts mortality in patients with moderate to severe COPD and normal LVEF. TR predicts exercise capacity after adjustment for lung function parameters. COPD severity and hyperinflation are associated with increased heart rate and reduced stroke volume, likely due to reduced preload to a left ventricle. Our findings emphasize that heart function parameters should be investigated in COPD. Pulsed wave tissue Doppler indices of systolic and diastolic heart function in COPD did not predict exercise capacity or mortality, however diastolic mitral Doppler data in COPD could be interpreted as a sign of low LV preload and not necessarily an intrinsic impairment in LV relaxation/compliance.

## Competing interests

The authors declare that they have no competing interests.

## Authors’ contributions

MMS made substantial contributions to conception, design, acquisition of data, analysis and interpretation of data and drafting the manuscript. MD, JK, IS, KKI made substantial contributions to conception, design, acquisition of data and revised it critically for important intellectual content. DM, SGJ made substantial contributions to patient enrollment and acquisition of data. All authors read and approved the final manuscript.

## Pre-publication history

The pre-publication history for this paper can be accessed here:

http://www.biomedcentral.com/1471-2261/13/84/prepub
